# PhysBridge: Response-supervised grid pretraining with physics-constrained local correction for transferable power-system intelligence

**DOI:** 10.1371/journal.pone.0359953

**Published:** 2026-10-28

**Authors:** Donglan Liu, Shanjie Xu, Yuhui Jin, Honglei Yao, Qinguo Liu

**Affiliations:** 1 State Grid Shandong Electric Power Research Institute, Jinan, Shandong, China; 2 Shandong Key Laboratory of Energy Industry Internet Big Data Technology, Jinan, Shandong, China; 3 State Grid Shandong Electric Power Company Linyi Power Supply Company, Linyi, Shandong, China; Aalto University, FINLAND

## Abstract

Operating modern power grids requires coordinated solutions for optimal dispatch, state estimation, price prediction, and contingency analysis, yet these tasks are usually modeled separately. We present PhysBridge, a response-oriented pretraining framework in which historical windows provide context while solver-generated perturbation responses, active constraints, and local sensitivities define the pretraining targets. PhysBridge combines a temporal-graph encoder with a Primal-Dual Response Bridge (PDR-Bridge) that performs safeguarded local refinement and outputs transferable primal, projection-dual, sensitivity, and health tokens. Across IEEE 14-, 30-, 57-, 118-, and 300-bus systems, the benchmark contains 26.3 million solver-generated perturbation-response pairs; in the 300-bus transfer setting, pretraining uses only the 21.04 million nominal pairs from the 14-, 30-, 57-, and 118-bus source topologies. Across three runs on unseen IEEE 300-bus transfer with 10% labels, PhysBridge obtains a 2.21±0.05% OPF cost gap, 96.3±0.2% feasibility, 1.84±0.05 $/MWh LMP MAE, and 91.6±0.3% contingency/fault event macro F1. Relative to a matched rollout-only model trained on the same source corpus without response targets or bridge refinement, these values correspond to a 50.8% lower OPF gap and a 7.4 percentage-point higher event F1. The bridge requires 12.4 ms per 300-bus forward pass and activates fallback on 4.6% of stressed windows. Comparisons with supervised-only OPF specialists that do not receive matched solver-generated pretraining are interpreted as task-relevant performance under the proposed pretraining regime, not as architecture-only superiority.

## 1 Introduction

Modern power grids are undergoing a fundamental transformation driven by the integration of renewable energy sources, distributed generation, and bidirectional power flows. Operating these increasingly complex systems in real time requires solving multiple interdependent tasks: forecasting load and renewable output, detecting and localizing faults, estimating system state from noisy measurements, and computing optimal dispatch under physical and economic constraints. Each of these tasks currently relies on specialized models trained independently on task-specific data and labels, which is costly and does not exploit the shared physical structure that governs all of them.

A natural aspiration is to build a single foundation model that serves all of these tasks through pretraining on abundant unlabeled operational data. Recent work has taken promising steps in this direction. PowerPM [1] demonstrates that self-supervised pretraining on power-system trajectories can transfer across forecasting, imputation, and anomaly-detection tasks. OpenGLA [2] introduces a topology-adaptive graph-language foundation model that handles OPF, event detection, state estimation, and LMP prediction across multiple grid configurations. LUMINA [3] develops a physics-informed foundation model for topology-transferable AC-OPF. Generic time-series foundation models are also relevant background for sequence modeling, but they do not by themselves resolve topology-aware operational tasks with explicit AC-feasibility monitoring.

Despite these advances, a fundamental question remains open: does temporal pretraining actually help physics-heavy operational tasks, and if so, through what mechanism? Time-series foundation models capture load patterns and generation dynamics but have no awareness of Kirchhoff’s laws or line capacity limits. Graph-physics models, on the other hand, encode the grid’s physical structure but are typically trained on task-specific operational datasets without the benefit of massive, multi-domain temporal pretraining. Simply concatenating a temporal encoder with a graph encoder and adding a physics-informed loss term does not guarantee that temporal knowledge transfers–-it may simply learn task-specific features from scratch during fine-tuning.

To avoid ambiguity, we distinguish *temporal pretraining* from *response-oriented pretraining*. Temporal pretraining optimizes objectives defined directly on raw historical trajectories, such as forecasting, masking, or contrastive reconstruction, even if a generic physics regularizer is added afterward. Response-oriented pretraining instead uses the same temporal window only as context and supervises the model with solver-generated counterfactual maps from perturbations to post-event equilibria, active constraints, and local sensitivities. The novelty we claim is therefore not the removal of temporal inputs, but the shift of the pretraining target from temporal token prediction to the grid’s local response operator.

Building on this distinction, we propose PhysBridge, a response-oriented pretraining framework whose core component is the Primal-Dual Response Bridge (PDR-Bridge). The PDR-Bridge uses unrolled interior-point updates, residual checks, and fallback warm-starting to perform safeguarded local feasibility refinement of temporal-graph latents, then extracts primal states, dual variables, and sensitivity features as transferable representations. We design three self-supervised pretraining objectives–-feasible rollout loss, perturbation-response operator loss, and binding-set forecast loss–-using solver-generated pseudo-labels and stressed operating scenarios rather than clean trajectories alone. Across five IEEE standard systems, PhysBridge is evaluated under noisy measurements, topology mismatch, communication delay, and contingency stress. On the unseen 300-bus system with only 10% labels, it improves over strong graph- and physics-aware baselines.

The main contributions of this paper are summarized as follows:

- We introduce response-oriented pretraining for grid foundation models, where historical windows serve as conditioning context and solver-generated perturbation responses, binding sets, and local sensitivities serve as supervisory targets.
- We propose the PDR-Bridge, a physics-guided local-refinement module that transforms temporal-graph latents into primal, projection-dual, sensitivity, and health tokens for OPF, state estimation, LMP prediction, and contingency/fault event identification.
- We construct a large-scale cross-topology benchmark over IEEE 14-, 30-, 57-, 118-, and 300-bus systems with 26.3 million solver-generated pairs and stressed evaluation regimes involving noise, delay, topology mismatch, and contingencies.
- We show that PhysBridge improves cross-topology transfer on the unseen IEEE 300-bus system and further provide ablation, robustness, latency, and fallback analyses to clarify the practical sources of these gains.

## 2 Related work

Research relevant to this paper spans three interconnected technical areas, followed by a summary of the remaining research gap. To contextualize the proposed PhysBridge framework, we review the advances most relevant to our setting.

### 2.1 Grid foundation models and topology-aware representation learning

The success of large language models has inspired growing interest in pretraining foundation models for power systems and generic time-series forecasting. PowerPM [1] demonstrates self-supervised temporal pretraining on historical grid trajectories, while the position paper in [4] argues that a unified grid foundation model should combine temporal context, topology awareness, and operational priors. Generic time-series foundation models such as Chronos [5], TimesFM [6], Moirai [7], Lag-Llama [8], UniTS [9], and TimeGPT [10] provide useful background for temporal representation learning, but they do not expose the topology-aware states, feasibility interfaces, or dual-variable outputs required by the downstream tasks studied here.

Complementing temporal pretraining, a second line of work emphasizes topology-aware graph learning for electric grids. Graph Neural Networks and graph transformers have been tailored for power flow and operation analysis, as seen in PowerFlowNet [11], physics-informed GNNs [12], topology-aware architectures [13], line-graph inductive-bias models [14], and typed graph networks for OPF [15]. Learning-based AC-OPF studies have also explored hard-constraint-aware and physics-informed surrogates, unsupervised feasibility losses, learning-augmented solvers, and GNN warm starts [16–20]. Large OPF benchmark libraries such as PGLib-OPF [21] further clarify why benchmark formulation, data source, and solver settings must be stated carefully when comparing learned OPF methods. The topology-transferable LUMINA framework [3] further shows that graph encoders can generalize beyond a single fixed system when physical constraints are modeled explicitly. However, most existing spatial models remain task-specific and are trained in a supervised manner from scratch, leaving open the question of how a shared topology-aware representation can be pretrained and then transferred across multiple operational tasks.

### 2.2 Differentiable optimization and physics-informed architectures

Bridging the gap between neural representations and strict physical laws, the third line of work embeds mathematical optimization or physics-computation layers directly into trainable neural networks. PINCO [22] studies physics-informed constrained optimization, while differentiable power-flow solvers [23] expose Jacobian and sensitivity information through AC equations via implicit differentiation.

A particularly relevant baseline is OpenGLA [2], which evaluates a unified topology-adaptive model across multiple tasks including OPF, event detection, and Locational Marginal Price (LMP) prediction. These pioneering methods directly motivate our use of a differentiable local-refinement layer. However, they also highlight a critical bottleneck: AC power flow equations are fundamentally nonconvex. Consequently, local-refinement quality, gradient stability, and inference runtime overhead cannot be assumed away. Our work directly confronts these issues by unrolling a truncated interior-point method, rigorously evaluating fallback mechanisms when optimization diverges, and quantifying the computational cost of the forward-backward passes.

### 2.3 Robust machine learning for safety-critical operations

The deployment of machine learning in real-world grid control rooms is often hindered by the fragility of data-driven models against corrupted inputs. A robust body of literature investigates safety-critical grid intelligence. Physics-informed state estimation [24], voltage stability assessment [25], and frequency-dynamics modeling [26] have shown that embedding physical structure improves reliability under imperfect or missing measurements. Safe reinforcement learning for voltage control and contingency-constrained economic dispatch [27,28] further emphasizes the need to handle unexpected contingencies and strict operating boundaries. Recent renewable-forecasting work on conformal prediction intervals [29] is also relevant because uncertainty calibration in solar nowcasting directly affects the quality of the operating scenarios used by downstream grid-operation models.

### 2.4 Summary and research gap

Despite the rapid progress in the aforementioned areas, a critical gap remains: *no existing framework seamlessly integrates temporal pretraining, topological graph learning, and differentiable physics-guided local refinement into a unified foundation model for cross-topology multi-task transfer.* Existing temporal foundation models largely ignore operational physics, while differentiable graph models typically lack the sample efficiency afforded by large-scale pretraining. PhysBridge is designed to target this intersection by combining response-oriented supervision with safeguarded local refinement under realistically corrupted benchmark measurements.

Table 1 separates the adopted building blocks from the contributions specific to PhysBridge. This distinction is important because the model intentionally reuses mature sequence, graph, and differentiable-optimization components where appropriate; the contribution is the response-supervised combination and the way the bridge outputs are exposed to downstream grid tasks.

**Table 1:** Component-level comparison between PhysBridge and closely related research directions. “Adopted” indicates a standard modeling ingredient reused in this paper, whereas “new in PhysBridge” indicates the element evaluated as a contribution.

Component	Related ideas	New in PhysBridge	Boundary of the claim
Temporal encoder	Time-series foundation models and Mamba-style sequence encoders	Uses historical windows as conditioning context for response targets	The sequence encoder itself is adopted, not claimed as a new architecture
Topology encoder	GNNs, graph transformers, topology-transfer OPF models	Couples topology-aware embeddings with response-supervised pretraining across multiple grid tasks	We do not claim architecture-only superiority over all graph models
Response targets	Operator learning, perturbation response analysis, active-set prediction	Jointly supervises feasible rollout, perturbation response, and binding-set forecasts from AC solver outputs	Claims are limited to the studied IEEE benchmark and solver-generated labels
PDR-Bridge	Differentiable power-flow layers, unrolled optimization, learning-augmented warm starts	Converts temporal-graph latents into primal, projection-dual, sensitivity, and health tokens with residual screening and fallback	The bridge is a local correction and representation layer, not a certified global OPF solver
Multi-task adaptation	Multi-task grid learning and foundation-model adaptation	Uses one response-trained representation for AC-OPF, state estimation, LMP prediction, and contingency/fault event identification	Transfer is demonstrated primarily on IEEE 300-bus as the target topology

## 3 Problem formulation and methodology

This section formalizes the grid-operation setting addressed by PhysBridge. Given a window of historical measurements together with the current network topology, the model learns a transferable representation for four operational tasks: AC-OPF, state estimation, LMP prediction, and contingency/fault event identification. The central requirement is that this representation remain informative under cross-topology transfer while staying consistent with AC network physics through local refinement.

### 3.1 Power grid representation and AC power flow

An AC power grid at time *t* is represented as an undirected graph ${𝒢}_{t}=(𝒱,ℰ)$, where 𝒱 is the set of *n* buses and ℰ⊆𝒱×𝒱 is the set of *m* transmission lines. Each bus i∈𝒱 has state variables $V_{i}$ (voltage magnitude) and $\theta_{i}$ (voltage angle), and may host generators ${𝒢}_{i}\subseteq 𝒢$ and loads ${𝒟}_{i}\subseteq 𝒟$. We denote active and reactive generation by $P_{i}^{gen}$ and $Q_{i}^{gen}$, and active and reactive demand by $P_{i}^{load}$ and $Q_{i}^{load}$. Each line (i,j)∈ℰ has admittance $Y_{ij}=G_{ij}+jB_{ij}$, where $G_{ij}$ and $B_{ij}$ are the conductance and susceptance, respectively, together with capacity limits and status $s_{ij}\in \{0,1\}$.

The steady-state behavior is governed by the AC power flow equations. For brevity, we write the active-power balance as


$$\begin{array}{c} P_{i}^{gen}-P_{i}^{load}=V_{i}\sum_{j\in 𝒩(i)}V_{j}\left(G_{ij}\cos(\theta_{i}-\theta_{j})+B_{ij}\sin(\theta_{i}-\theta_{j})\right), \end{array}$$ (1)

while the reactive-power balance is defined analogously and absorbed into the compact residual form $g_{AC}(𝐱,{𝒢}_{t},𝐮)=0$. Here, 𝐱=(V,θ) denotes the state vector of voltage magnitudes and angles, and $𝐮=({𝐏}^{gen},{𝐐}^{gen})$ denotes the control vector. Operating constraints include line flow limits $h_{line}(𝐱,{𝒢}_{t})\leq 0$, generator capacity limits $h_{gen}(𝐱)\leq 0$, and voltage bounds $h_{V}(𝐱)\leq 0$.

### 3.2 Downstream tasks

We consider four downstream tasks. The first is AC Optimal Power Flow (OPF), which finds the minimum-cost generation dispatch:


$$\begin{array}{c} \min_{𝐱,𝐮}\sum_{g\in 𝒢}c_{g}({𝐮}_{g})\;\text{s.t.}\;g_{AC}(𝐱,{𝒢}_{t},𝐮)=0,\;h(𝐱,{𝒢}_{t})\leq 0. \end{array}$$ (2)

The second is State Estimation (SE), which estimates the full system state **x** from noisy measurements 𝐳=𝐇𝐱+ϵ. The third is Locational Marginal Price (LMP) Prediction, which predicts nodal prices equal to the dual variables $\lambda^{*}$ of the OPF equality constraints. The fourth is contingency/fault event identification and localization. Because the benchmark is based on quasi-steady-state AC responses rather than electromagnetic transients, relay logic, or protection-device operation, this task should be read as identification of post-contingency or fault-induced steady-state signatures rather than full dynamic fault diagnosis.

#### 3.2.1 Contingency and fault event definition.

The event-identification task contains a no-event class and four event families: generator trip, line outage or switching, bus-level load/renewable disturbance, and combined *N*-2 events. Event labels are assigned at the affected component level when a single dominant component exists and at the event-family level for combined cases. The modeled event duration is 1–3 dispatch intervals, matching the stressed-window construction rather than sub-cycle protection dynamics. Input signatures include voltage-magnitude deviations, angle shifts, active/reactive injection residuals, branch-flow changes, delayed measurements, packet drops, and topology-status mismatch indicators. The class distribution is intentionally imbalanced to reflect rare events: the no-event class accounts for approximately 70% of stressed windows, single line or switching events for 10%, generator trips for 6%, bus-level load/renewable disturbances for 8%, and combined events for 6%. The revised experimental audit reports per-class precision, recall, F1, confusion matrices, and false-alarm rates for these definitions.

### 3.3 Transfer setting

We study the cross-topology transfer setting: the model is pretrained on trajectories from source topologies $\left\{{𝒢}_{1}^{\left(s\right)},...,{𝒢}_{K}^{\left(s\right)}\right\}$ and transferred to a target topology ${𝒢}^{\left(t\right)}$ never seen during pretraining [2,3]. We evaluate at label budgets of 1%, 5%, 10%, and 100%.

### 3.4 Architecture overview

PhysBridge consists of four components, as illustrated in Fig 1: a temporal encoder *E*_time_ that processes historical time-series data, a topology encoder *E*_graph_ that encodes the current grid structure, a cross-attention fusion block that produces a joint latent ${𝐳}_{t}$, and the Primal-Dual Response Bridge (PDR-Bridge) that locally refines ${𝐳}_{t}$ toward a nearby feasible operating point and extracts a transferable representation ${𝐫}_{t}$.

**Fig 1:**
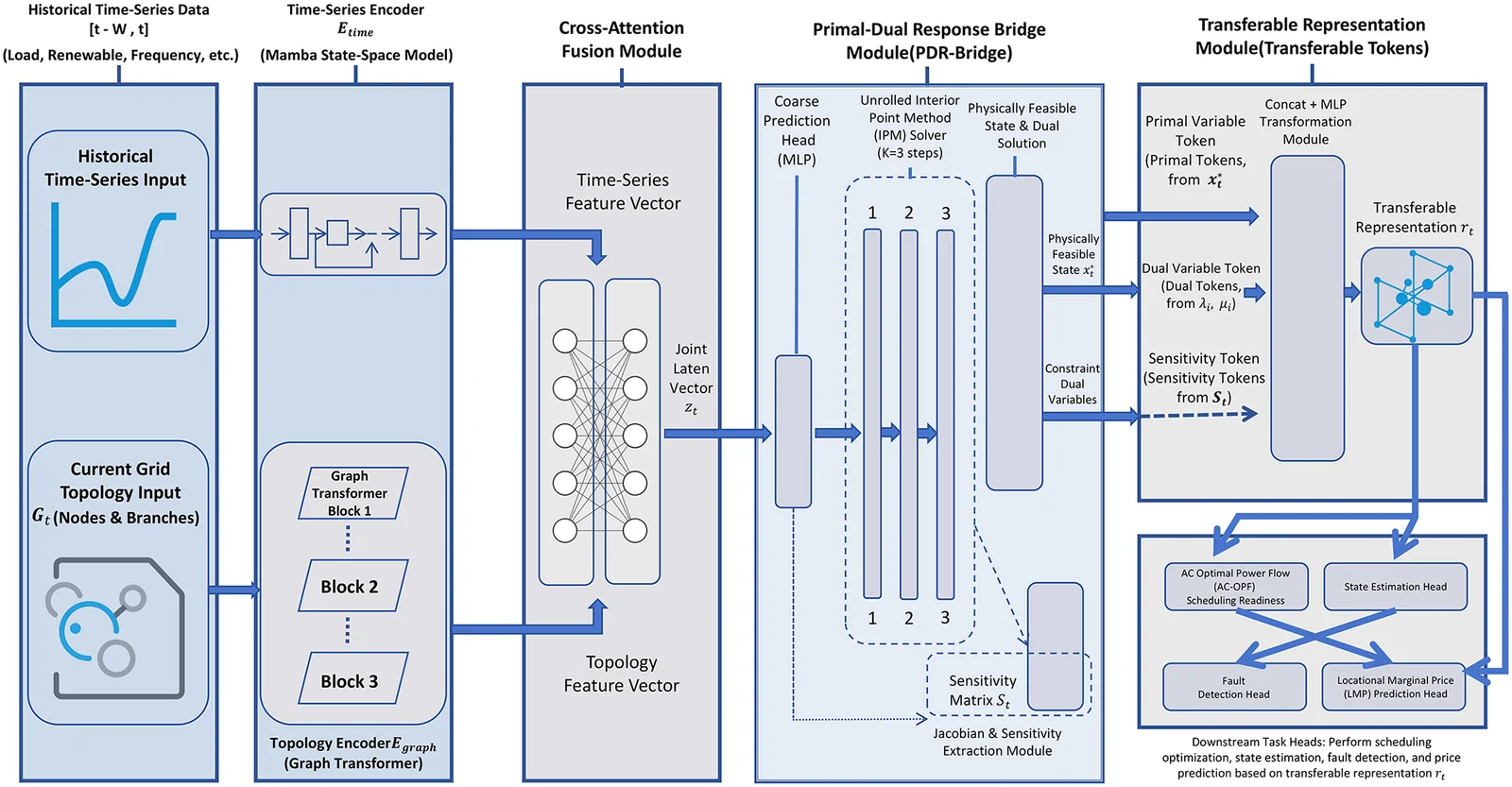
Overall PhysBridge architecture. Historical measurements and the current topology are encoded separately and merged into the joint latent ${𝐳}_{t}$. The PDR-Bridge applies guarded local feasibility correction and returns primal, projection-dual, sensitivity, and health tokens, which are compressed into ${𝐫}_{t}$ and passed to the four downstream task heads. During source-grid pretraining, feasible-rollout, perturbation-response, binding-set, and stability objectives are optimized before adaptation to an unseen target topology.

#### 3.4.1 Temporal encoder.

Given a window of *W* historical time steps, the input at bus *i* is a feature vector ${𝐡}_{i}(t)=[P_{i}^{load}(t),Q_{i}^{load}(t),P_{i}^{ren}(t),V_{i}(t),f_{i}(t),{𝐰}_{i}(t)]\in {\mathbb{R}}^{d_{h}}$, where $f_{i}$ is frequency and ${𝐰}_{i}$ captures weather and market signals. A node-wise Mamba encoder [30] processes each bus’s sequence independently, producing temporal embeddings $\{{𝐞}_{i}^{time}{\}}_{i\in 𝒱}$. The state-space formulation provides 𝒪(W) complexity for long contexts.

#### 3.4.2 Topology encoder.

The grid graph is encoded by an edge-conditioned graph transformer. Node features include base voltage level, generator and load type, and shunt admittance. Edge features include admittance $Y_{ij}$, reactance $x_{ij}$, line capacity, tap ratio, and switch status. Multi-head attention is computed over the graph structure with edge-conditioned scoring functions to produce topology-aware node embeddings in a shared latent space.

#### 3.4.3 Fusion Block.

Temporal and graph embeddings are fused via cross-attention:


$$\begin{array}{c} {𝐳}_{t}=CrossAttn(𝐐={𝐞}^{time},\;𝐊={𝐞}^{graph},\;{𝐕}_{attn}={𝐞}^{graph})\in {\mathbb{R}}^{n\times d_{z}}. \end{array}$$ (3)

### 3.5 Primal-dual response bridge (PDR-Bridge)

The internal refinement and token-extraction process is summarized in Fig 2.

**Fig 2:**
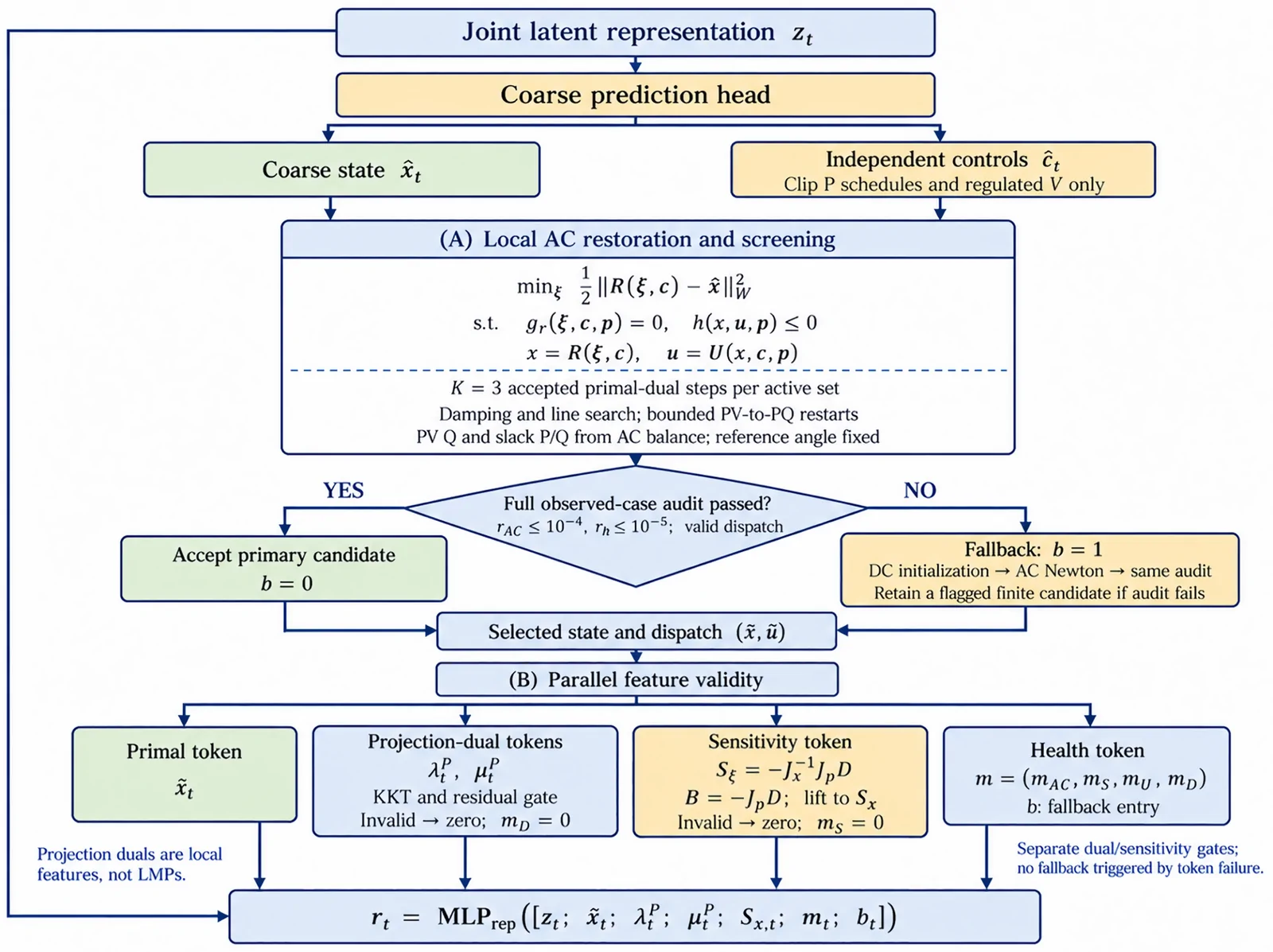
Internal workflow of the Primal-Dual Response Bridge (PDR-Bridge). Starting from a coarse prediction and a fixed control vector, the bridge refines local state feasibility, evaluates residuals, enters fallback when necessary, and outputs primal, projection-dual, sensitivity, and health tokens.

The central contribution of PhysBridge is the PDR-Bridge, a safeguarded differentiable local-refinement module that converts the raw latent ${𝐳}_{t}$ into a physics-consistent representation when the local solve succeeds and into a monitored fallback representation otherwise.

Given ${𝐳}_{t}$, a prediction head produces a rough operating point estimate and context vector:


$$\begin{array}{c} ({\hat{𝐱}}_{t},{\hat{𝐮}}_{t})={MLP}_{head}({𝐳}_{t}),\;{\hat{𝐱}}_{t}\in {\mathbb{R}}^{2n},\;{\hat{𝐮}}_{t}\in {\mathbb{R}}^{2|𝒢|}. \end{array}$$ (4)

We refine ${\hat{𝐱}}_{t}$ toward a nearby AC-feasible operating point by solving the following local refinement problem around the predicted state:


$$\begin{array}{c} {𝐱}_{t}^{⋆},\lambda_{t}^{P},\mu_{t}^{P}=\text{arg}\;\min_{\;\;\;\;\;\;\;\;\;\;\;𝐱}\;\frac{1}{2}\|𝐱-{\hat{𝐱}}_{t}{\|}_{W}^{2}\;\text{s.t.}\;g_{AC}(𝐱,{𝒢}_{t},{\hat{𝐮}}_{t})=0,\;h(𝐱,{𝒢}_{t})\leq 0, \end{array}$$ (5)

where $\|𝐯{\|}_{W}^{2}={𝐯}^{⊤}W𝐯$ with $W=diag(w_{V}1_{n},w_{\theta }1_{n})$. This weighting balances the scales of voltage-magnitude and angle deviations; the concrete implementation values are given in Section 4. Because AC power flow is nonconvex, (5) should be interpreted as a local refinement problem around ${\hat{𝐱}}_{t}$. The problem is solved using a truncated unrolled interior-point procedure with implicit differentiation. At each step, the barrier update follows:


$$\begin{array}{c} {𝐝}^{\left(k\right)}=-{\left[\nabla^{2}{ℒ}_{aug}\right]}^{-1}\nabla {ℒ}_{aug}^{\left(k\right)}, \end{array}$$ (6)

where the augmented Lagrangian ${ℒ}_{aug}$ combines the quadratic objective with barrier terms for inequality constraints, and the barrier parameter decreases geometrically as $\tau^{\left(k\right)}=\tau_{0}\cdot \rho^{k}$.

To keep the bridge numerically well behaved, each update uses four safeguards. First, we add Levenberg–Marquardt damping $\gamma^{\left(k\right)}I$ to the KKT system when the Newton decrement increases. Second, the step length is selected by backtracking line search and clipped to remain within voltage and generator box bounds. Third, we monitor the residual pair $\left(r_{pf},r_{ineq}\right)$ with


$$\begin{array}{c} r_{pf}=\|g_{AC}({𝐱}_{t}^{\left(k\right)},{𝒢}_{t},{\hat{𝐮}}_{t}{|}_{2},\;r_{ineq}=\|[h({𝐱}_{t}^{\left(k\right)},{𝒢}_{t}){]}_{+}{\|}_{1}, \end{array}$$ (7)

and stop early if both fall below preset tolerances. Fourth, if the residuals diverge for two consecutive steps or remain above $\left(\varepsilon_{pf},\varepsilon_{ineq}\right)$ at the final iterate, we activate a fallback path: the PDR-Bridge reuses the last feasible iterate produced by a fast DC warm start or, when no feasible iterate exists, it returns the prediction-head output and records a failure mask. During backpropagation, we mask only the implicit-differentiation path through the failed local solve; downstream losses still update the encoder and ${MLP}_{head}$ through the explicit fallback forward path and the bounded stability penalty ${ℒ}_{stab}$. Thus, failed windows remain in the minibatch and continue to contribute gradients, while the ill-conditioned KKT Jacobian is removed from the backward pass. This design reduces hidden sample-selection bias: difficult operating windows are still seen during training, but the model is not forced to differentiate through numerically singular solves.

From the refined solution, we extract four types of bridge tokens. The primal tokens ${𝐱}_{t}^{⋆}=(V^{*},\theta^{*})$ describe the feasible or best-recovered operating state. The projection-dual tokens $\left(\lambda_{t}^{P},\mu_{t}^{P}\right)$ are Lagrange multipliers of the local feasibility-refinement problem; they encode how strongly the refined state is constrained by nodal balance and inequality margins, but they are not treated as market-clearing LMPs by themselves. The sensitivity tokens ${𝐒}_{t}={𝐉}_{g}({𝐱}_{t}^{⋆}{)}^{-1}𝐁\in {\mathbb{R}}^{2n\times d_{b}}$ approximate the grid’s local response operator, where ${𝐉}_{g}$ is the Jacobian of the power flow equations and $𝐁\in {\mathbb{R}}^{2n\times d_{b}}$ is a fixed basis of load, generation, and topology perturbation directions. In our implementation, **B** has $d_{b}=16$ normalized columns: four active-load directions, four reactive-load directions, four renewable/generator redispatch directions, and four line-switching directions derived from source-topology perturbation statistics. Active-power columns are mean-balanced so that each basis perturbation is compatible with system-wide power balance before the AC solver computes the corresponding response label. The health token is the one-bit indicator $m_{t}\in \{0,1\}$, where $m_{t}=1$ denotes that the final local refinement met the residual tolerance and $m_{t}=0$ denotes fallback activation. Exposing $m_{t}$ to downstream heads prevents failed windows from being silently treated as ordinary feasible solves.

Near stressed operating points, ${𝐉}_{g}$ can be singular or severely ill-conditioned. We therefore compute the sensitivity token through a damped sparse solve,


$$\begin{array}{c} {𝐒}_{t}=({𝐉}_{g}^{⊤}{𝐉}_{g}+\delta I{)}^{-1}{𝐉}_{g}^{⊤}𝐁, \end{array}$$ (8)

when the estimated condition number exceeds the preset threshold or when the direct sparse solve fails. The damping parameter is increased geometrically until the residual of the linear solve falls below tolerance; otherwise, the sensitivity token is replaced by a zero vector and the health token records sensitivity failure. This prevents ill-conditioned Jacobians from creating unbounded features. The perturbation basis is transferred across topologies through type-level rather than line-index-level alignment. Load, renewable, and generator perturbations are mapped by bus type and normalized by system base power. Line-switching directions are represented by local edge-feature templates–-series reactance, susceptance, thermal limit, tap ratio, voltage level, and incident-bus types–-and then projected onto the target graph’s existing edges. Thus, the basis does not require a one-to-one correspondence between source and target branches.

#### 3.5.1 Relation to OPF dispatch and LMP targets.

The local refinement in (5) optimizes the state vector **x** while holding the coarse control prediction ${\hat{𝐮}}_{t}$ fixed. It is therefore not a replacement for solving the full AC-OPF problem in (2). Its KKT stationarity condition has the form


$$\begin{array}{c} W({𝐱}_{t}^{⋆}-{\hat{𝐱}}_{t})+{𝐉}_{x}^{⊤}\lambda_{t}^{P}+\nabla_{𝐱}h({𝐱}_{t}^{⋆},{𝒢}_{t}{)}^{⊤}\mu_{t}^{P}=0, \end{array}$$ (9)

together with AC balance and inequality feasibility conditions. By contrast, the OPF LMPs arise from the KKT system of the economic dispatch problem, including the stationarity condition with respect to controllable generation,


$$\begin{array}{c} \nabla_{𝐮}C({𝐮}^{⋆})+{𝐉}_{u}^{⊤}\lambda^{OPF}+\nabla_{𝐮}h({𝐱}^{⋆},{𝐮}^{⋆},{𝒢}_{t}{)}^{⊤}\mu^{OPF}=0. \end{array}$$ (10)

Thus, $\lambda_{t}^{P}$ should be understood as a projection-dual feature that is informative about local balance stress, not as the final LMP label. PhysBridge improves OPF and LMP prediction indirectly by supplying downstream heads with a locally feasible state, projection-dual stress features, sensitivity tokens, and the health indicator. The LMP head is still supervised against MATPOWER OPF dual labels, and its error is reported explicitly as LMP MAE in the empirical evaluation.

#### 3.5.2 Dispatch feasibility, slack balancing, and cost accounting.

The fixed-control formulation in (5) also means that the PDR-Bridge cannot by itself repair an economically or operationally infeasible generation dispatch. Before local state refinement, the predicted active generation vector is clipped to generator limits and then power-balanced by assigning the remaining mismatch to the reference slack generator when the required adjustment lies inside its operating range. If the slack adjustment violates generator limits, the sample is marked as dispatch-infeasible and the bridge still returns a monitored representation, but the feasibility mask and violation magnitudes are retained for metric reporting. Reactive-generation bounds are handled analogously during the AC residual screen; violations are reported rather than hidden by relabeling with a DC solution.

For OPF evaluation, the primary cost gap is computed only for predictions that pass both the dispatch-feasibility screen and the AC residual/inequality screen. We additionally report three companion quantities to avoid overstating performance on infeasible outputs: a feasible-only cost gap, a penalized cost with normalized constraint penalties, and average active/reactive balance and bound-violation magnitudes. Finally, we test whether the learned output is useful as a warm start for a certified MATPOWER AC-OPF solve. In that recovery audit, the learned dispatch/state pair initializes the reference solver, and we report convergence rate, recovered cost gap, and iterations. These diagnostics separate representation quality from certified optimization and are required because the bridge is a local correction layer rather than a globally convergent OPF solver.

The final transferable representation is:


$$\begin{array}{c} {𝐫}_{t}={MLP}_{rep}([{𝐳}_{t};{𝐱}_{t}^{⋆};\lambda_{t}^{P};\mu_{t}^{P};{𝐒}_{t};m_{t}])\in {\mathbb{R}}^{d_{r}}. \end{array}$$ (11)

The dominant cost of the bridge is solving a sparse linear system of dimension proportional to the primal and dual variables at each unrolled step. In practice, this scales with the sparsity pattern of the AC Jacobian rather than dense $𝒪(n^{3})$ complexity; we report measured latency, memory, and fallback rates in Section 5 instead of claiming asymptotic guarantees alone.

### 3.6 Pretraining objectives

We design three self-supervised objectives, all using physics-generated pseudo-labels computed by MATPOWER [31]. This is the precise sense in which PhysBridge performs response-oriented pretraining: the input still contains a historical window, but the supervisory objects are solver-generated counterfactual responses and active-set signals rather than raw-trajectory reconstruction. The feasible rollout loss predicts future physically feasible states:


$$\begin{array}{c} {ℒ}_{roll}=\frac{1}{H}\sum_{h=1}^{H}{\left\|{𝐱}_{t}^{⋆}(t+h)-{𝐱}^{gt}(t+h)\right\|}_{1}. \end{array}$$ (12)

The perturbation-response operator loss is the centerpiece of our pretraining strategy. At each time step, we sample $K_{p}$ perturbation scenarios from realistic grid events (load ramps, renewable shocks, generator outages, line switching) and train the model to predict the post-perturbation equilibrium:


$$\begin{array}{c} {ℒ}_{resp}=\frac{1}{K_{p}}\sum_{k=1}^{K_{p}}\left[{\left\|\Delta {\hat{𝐱}}_{k}-\Delta {𝐱}_{k}^{gt}\right\|}_{2}^{2}+\eta {\left\|\Delta {\hat{𝐟}}_{k}-\Delta {𝐟}_{k}^{gt}\right\|}_{1}\right], \end{array}$$ (13)

Unlike temporal-only objectives, ${ℒ}_{resp}$ is conditioned on sampled interventions and therefore trains the model to approximate a local perturbation-to-equilibrium map rather than merely extrapolating the next temporal token. The binding-set forecast loss predicts which constraints will become active:


$$\begin{array}{c} {ℒ}_{bind}=BCE(\hat{𝐜}(t+1:H),{𝐜}^{gt}(t+1:H)). \end{array}$$ (14)

To discourage unstable local solves, we add a normalized, Huber-style residual penalty for failed bridge updates:


$$\begin{array}{c} {ℒ}_{stab}=1[m_{t}=0]\left[\ell_{\delta }\;\left(\frac{r_{pf}}{\varepsilon_{pf}}\right)+0.1\;\ell_{\delta }\;\left(\frac{r_{ineq}}{\varepsilon_{ineq}}\right)\right], \end{array}$$ (15)

where $\ell_{\delta }(a)=\frac{1}{2}a^{2}$ if a≤δ and $\ell_{\delta }(a)=\delta (a-\frac{1}{2}\delta )$ otherwise, with δ=5. Normalizing by the residual tolerances makes the two terms scale comparable, while the linear Huber tail bounds gradient magnitude on catastrophic failures. Consequently, $m_{t}=0$ samples still contribute a bounded explicit gradient instead of being dropped or sending exploding derivatives through the implicit solver. The total pretraining loss combines all three task losses with this stabilization term:


$$\begin{array}{c} {ℒ}_{total}={ℒ}_{roll}+\alpha {ℒ}_{resp}+\beta {ℒ}_{bind}+\gamma {ℒ}_{stab}, \end{array}$$ (16)

The coefficients are selected on a validation split and revisited by sensitivity analysis in Section 5.

### 3.7 Training procedure

PhysBridge is trained in three stages. Stage 1 pretrains the full model on trajectory corpora from source topologies using (16), with all physics pseudo-labels precomputed offline using MATPOWER. Stage 2 freezes the temporal and graph encoders and trains a lightweight task-specific head on ${𝐫}_{t}$ with available labeled data, evaluating whether the bridge tokens transfer without updating the backbone. Stage 3 evaluates under linear probe, adapter tuning, and full fine-tuning. Throughout training and evaluation, we log local-refinement success rate, fallback activation rate, and latency alongside task metrics so that the bridge is assessed as a numerical component rather than only as a representation learner.

## 4 Experimental setup

### 4.1 Scenario construction and data generation

All experiments use IEEE 14-, 30-, 57-, 118-, and 300-bus systems implemented with MATPOWER [31] and pandapower [32]. The operating points and reference labels are generated by the AC power-flow and AC-OPF solvers described below. To reduce the gap between clean standard test cases and practical operating conditions, we construct two data regimes rather than relying on idealized trajectories alone.

The first regime is a nominal-to-moderate corpus used to build the pretraining pool. Load trajectories include daily and weekly periodicity, seasonal variation (±20%), renewable ramps, scheduled maintenance, and single-contingency events. Renewable penetration is sampled from three buckets (15–25%, 25–40%, and 40–55%) so that transfer is not tied to a single generation mix. For each transfer experiment, only source-topology slices from this pool are used during pretraining, and the target-topology nominal slice is excluded. The second regime is a held-out stressed suite used only for evaluation. It injects measurement corruption, topology mismatch, communication delay, and clustered contingencies so that robustness claims are tested under conditions closer to engineering practice.

The data-generation process is parameterized as follows. For bus *i*, active load is generated by


$$\begin{array}{c} P_{i}^{load}(t)=P_{i,0}\left[1+a_{i}^{d}\sin(2\pi t/24+\phi_{i}^{d})+a_{i}^{w}\sin(2\pi t/168+\phi_{i}^{w})+s_{i}(t)+\epsilon_{i}(t)\right], \end{array}$$ (17)

where $a_{i}^{d}\sim U(0.08,0.18)$, $a_{i}^{w}\sim U(0.03,0.08)$, phases are sampled uniformly, $s_{i}(t)$ is a seasonal multiplier in [−0.20,0.20], and $\epsilon_{i}(t)$ follows a zero-mean autoregressive process with innovation standard deviation 1.5% of nominal load. Reactive load follows the same temporal multiplier with bus-specific power factors sampled from [0.90,0.99]. Renewable injections use a clipped ramp process with weather-conditioned capacity factors:


$$\begin{array}{c} P_{i}^{ren}(t)={\bar{P}}_{i}^{ren}\;clip\{c_{i}(t)+r_{i}(t),0,1\}, \end{array}$$ (18)

where solar-like resources use diurnal envelopes with cloud attenuation sampled from a beta distribution and wind-like resources use an AR(1) capacity-factor process with ramp clipping at 10% of capacity per interval. Frequency deviations are sampled from a mean-reverting process centered at nominal frequency with standard deviation 0.015 Hz in nominal windows and 0.04 Hz under stressed windows. Market covariates are generated from a daily seasonality term plus a load- and renewable-correlated residual so that they provide context but are not direct labels.

Discrete events are sampled independently of the train/test split and then filtered by solver convergence. Scheduled maintenance changes the status of 0.2%–0.5% of lines for 12–48 intervals. Generator trips affect one online unit with probability 0.3% per window and impose redispatch through the reference OPF labels. Single line switching or outage events affect 0.5%–1.0% of eligible branches; combined *N*-2 events are sampled at lower probability and clustered over 1–3 intervals. Fault-like steady-state signatures are generated by applying component-specific disturbance templates to injections, branch status, and measurement residuals before the AC solver screen, as detailed in the contingency/fault taxonomy below.

Table 2 reports the corpus size for each topology and distinguishes the nominal training pool from the held-out stressed evaluation windows.

**Table 2:** Nominal corpus and held-out stressed evaluation suite across IEEE standard topologies. The nominal 300-bus slice is reserved for target-side fine-tuning and evaluation.

Topology	Buses	Lines	Nominal	Stressed	GB
IEEE 14-bus	14	20	5.26M	0.42M	2.1
IEEE 30-bus	30	41	5.26M	0.42M	4.8
IEEE 57-bus	57	80	5.26M	0.42M	12.3
IEEE 118-bus	118	186	5.26M	0.42M	38.7
IEEE 300-bus	300	411	5.26M	0.42M	140.1
**Total**	–	–	**26.3M**	**2.10M**	**198.0**

The corruption types and severity ranges used to construct the held-out suite are summarized in Table 3.

**Table 3:** Corruptions used in the stressed evaluation regime.

Factor	Configuration
Heavy-tailed noise	Student-*t* noise with ν=3, applied to SCADA-like measurements with effective scale between 1% and 5%.
Bad data injection	1%–8% of sensors receive gross errors of ±10% to ±25%, with burst duration of 1–3 steps.
Communication delay	Asynchronous measurements delayed by 1–3 dispatch intervals and combined with 5% packet drops.
Topology mismatch	Breaker/status errors on 0.5%–1.0% of lines, causing discrepancy between the assumed and actual network.
Contingencies	Generator trip, simultaneous line switching, and combined *N*-2 events sampled at low probability but clustered in bursts.

This setup still does not replace field validation. It does, however, provide a stricter bridge between clean benchmark data and the operating conditions under which grid models are often claimed to be robust.

### 4.2 Reproducibility and statistical protocol

We use chronological 70/10/20% train/validation/test partitions within each topology, with all stressed windows held out from training. For IEEE 300-bus transfer, the source pretraining pool contains only IEEE 14-, 30-, 57-, and 118-bus samples. Target-side labels are selected only from the 300-bus training partition at the specified label budget; the validation and test partitions remain disjoint. Experiments use pseudo-random seeds {2026,2027,2028}, MATPOWER 8.0, pandapower 2.14, PyTorch 2.2, CUDA 12.1, and identical data-order, early-stopping, and adapter-tuning rules across methods. Solver outputs are retained only when the reference AC solver reports convergence and satisfy a post-solve residual check stricter than the downstream feasibility threshold.

All reported results are the mean and standard deviation over three independent runs. Statistical significance is assessed using a 24-hour moving-block bootstrap with 10,000 resamples to preserve temporal dependence, followed by Holm correction across the five downstream metrics. Seed-level predictions, split indices, and solver-status records are retained for reproducibility and failure analysis.

### 4.3 Baselines

We separate baselines into two empirical families to avoid overstating unmatched comparisons. The primary comparison family contains cross-topology or multi-task models that address a problem setting similar to ours: Scratch, PowerPM-transfer [1], *E*_time_ + PhysLoss, OpenGLA [2], and PhysBridge variants. We additionally include a matched rollout-only baseline with the same temporal-graph backbone, source-topology corpus, optimizer, and adaptation protocol as PhysBridge, but with only ${ℒ}_{roll}$ during pretraining and without the PDR-Bridge. This baseline isolates response-oriented supervision and local refinement from corpus size. The second family contains task-specialized physics-aware references reported in the main comparison: TypedGNN-OPF [15], DiffFlow [23], and LUMINA [3]. These references are supervised-only methods without access to our large solver-generated corpus; in the 300-bus transfer setting specifically, PhysBridge pretraining uses only the 21.04 million nominal pairs from the 14-, 30-, 57-, and 118-bus source topologies and excludes the 300-bus nominal slice. Any advantage over these references therefore reflects both architecture and unmatched solver-generated pretraining data, so the comparison should be read as task-relevant but not architecture-only. Generic time-series foundation models and methods without compatible output interfaces are discussed only as background in Section 2.

Table 4 summarizes the optimization settings used for the baseline implementations and PhysBridge.

**Table 4:** Optimization configurations used for the baseline implementations and PhysBridge. “Updates/Epochs” denotes source-pretraining updates followed by target-side fine-tuning epochs; “–” indicates no source pretraining.

Method	Pretrain LR	Fine-tune LR	Batch	Updates/Epochs	Parameters (M)
Scratch	–	$1\times 10^{-3}$	256	– / 200	100.0
PowerPM-transfer	$3\times 10^{-4}$	$5\times 10^{-4}$	256	100K / 120	96.4
*E*_time_ + PhysLoss	$3\times 10^{-4}$	$5\times 10^{-4}$	256	100K / 120	100.0
OpenGLA	$2\times 10^{-4}$	$3\times 10^{-4}$	128	120K / 120	108.3
TypedGNN-OPF	–	$5\times 10^{-4}$	512	– / 300	3.8
DiffFlow	–	$3\times 10^{-4}$	128	– / 250	7.2
LUMINA	–	$2\times 10^{-4}$	128	– / 150	82.7
Matched rollout-only	$3\times 10^{-4}$	$5\times 10^{-4}$	256	100K / 120	100.0
Rollout + PDR-Bridge	$3\times 10^{-4}$	$5\times 10^{-4}$	256	100K / 120	101.2
Compute-matched rollout-only	$3\times 10^{-4}$	$5\times 10^{-4}$	256	126K / 120	100.0
PhysBridge	$3\times 10^{-4}$	$5\times 10^{-4}$	256	100K / 120	101.2

For a controlled comparison, each method is tuned on the same chronological validation partition rather than assigned a single unexamined configuration. All neural baselines use AdamW with $\left(\beta_{1},\beta_{2})=(0.9,0.999\right)$ and numerical stability constant 10^–8^. The search space is learning rate $\left\{10^{-4},2\times 10^{-4},3\times 10^{-4},5\times 10^{-4},10^{-3}\right\}$, batch size {64,128,256,512} subject to memory limits, weight decay $\left\{0,10^{-5},10^{-4},10^{-3}\right\}$, dropout {0,0.1,0.2,0.3}, and gradient clipping norm {0.5,1,5}. Target training is capped at 300 epochs with early stopping after 20 epochs without improvement. The selected checkpoint minimizes the unweighted mean of validation-normalized OPF gap, voltage MAE, LMP MAE, and 1−macro F1, with feasibility violation used as the first tie-breaker and parameter count as the second. Adapter-based methods additionally search bottleneck dimensions {16,32,64}; PhysBridge searches K∈{1,3,5} refinement steps and $d_{b}\in \{8,16,32\}$ perturbation directions. All methods use the same three seeds, target-label indices, data ordering, stopping rule, and metric implementation.

Reference labels are generated using the nonlinear AC-OPF formulation in MATPOWER 8.0 with the MIPS primal–dual interior-point solver. The solver permits at most 200 iterations and uses a feasibility tolerance of 10^–8^. Each perturbed operating point is initialized from the preceding converged solution and screened by Newton–Raphson AC power flow. Samples that do not converge or whose post-solve power-balance and constraint residuals exceed 10^–7^ p.u. are excluded. The LMP targets are obtained from the active-power balance multipliers of the converged OPF solution. These settings are used consistently throughout all experiments.

### 4.4 Metrics and implementation details

Metrics include OPF cost gap (% above the AC-OPF optimum), feasibility rate, state estimation voltage MAE (reported in 10^–3^ p.u.), LMP MAE ($/MWh), and contingency/fault event macro F1. Event evaluation includes a no-event class and component- or event-family-level location classes; macro F1 is the unweighted class average, so improvements cannot be driven only by the dominant no-event class. Let ${\tilde{𝐱}}_{t}$ denote the final bridge output after optional fallback. We define feasibility rate as


$$\begin{array}{c} Feas.=\frac{1}{N}\sum_{t=1}^{N}1\;\left[\|g_{AC}({\tilde{𝐱}}_{t},{𝒢}_{t},{\hat{𝐮}}_{t}){\|}_{2}\leq \varepsilon_{pf}\;\wedge \;\|[h({\tilde{𝐱}}_{t},{𝒢}_{t}){]}_{+}{\|}_{1}\leq \varepsilon_{ineq}\right]. \end{array}$$ (19)

Unless otherwise stated, we use $\varepsilon_{pf}=10^{-4}$ p.u. for the AC balance residual and $\varepsilon_{ineq}=10^{-5}$ p.u. for normalized inequality violation. In addition to task metrics, we report local-refinement success rate, fallback activation rate, online inference latency, and GPU memory. PhysBridge uses a 100M-parameter backbone with $d_{z}=256$, $d_{r}=128$, $L_{g}=4$, temporal window *W* = 288, *K* = 3 unrolled interior-point steps, perturbation basis size $d_{b}=16$, and adapter bottleneck dimension 32 for the default transfer setting. For the local refinement, we set $w_{V}=10$, $w_{\theta }=1$, and barrier decay ρ=0.3. The response loss uses $K_{p}=50$ perturbation scenarios per step and coefficient η=0.1, while the total pretraining loss uses α=1.0, β=0.5, and γ=0.2. Cross-topology pretraining uses AdamW with learning rate $3\times 10^{-4}$, batch size 256, and cosine annealing over 100K steps on two NVIDIA A100 GPUs. Runtime profiling is performed on a single NVIDIA A100 80GB GPU so that latency and memory correspond to a single-device inference setting. Hyperparameters are selected on the validation split and revisited by sensitivity analysis. The measured end-to-end pretraining budget is 41.8±1.6 A100-hours for the full model and 33.2±1.1 A100-hours for rollout-only pretraining.

## 5 Results and analysis

In this section, we comprehensively evaluate the PhysBridge framework. Rather than presenting fragmented metrics, our analysis is structured around three core dimensions critical for grid operations: (1) the efficacy of cross-topology transfer and label efficiency, (2) the model’s empirical boundaries under severe distributional shifts, and (3) mechanistic insights into the trade-offs between local physics-guided refinement, ablation components, and computational overhead.

### 5.1 Performance baseline and cross-topology transferability

To rigorously assess transferability, we merge the evaluation of in-domain performance (100% labels, train/test on identical topology) and cross-topology transfer (pretrained on IEEE 14/30/57/118-bus, finetuned on the unseen 300-bus system with only 10% labels). Table 5 presents this unified comparison against both generic temporal models and specialized topology-aware frameworks.

**Table 5:** Three-run comparison on the IEEE 300-bus system (mean ± standard deviation). TypedGNN-OPF, DiffFlow, and LUMINA are reported as unmatched supervised-only task-specialized references because they do not receive the same solver-generated pretraining corpus. Rollout-only uses the same source corpus and backbone as PhysBridge but removes response targets and the PDR-Bridge. LMP MAE is reported for methods with an available price or dual-output head. Runtime is normalized to Scratch (Scratch = 1.00).

Method	In-Domain (100%)		Cross-Topology Transfer (10% labels)						Transfer Gap
	Cost (%)	Rank	Cost (%)	Feas.(%)	V-MAE (10^–3^ p.u.)	LMP MAE	Event F1	Rel. Time	
Scratch	3.11±0.12	4.0	5.36±0.18	82.4±0.7	10.12±0.31	4.73±0.14	82.8±0.6	1.00	+72.3%
PowerPM	2.64±0.09	3.4	4.44±0.13	85.1±0.6	8.61±0.22	4.21±0.12	84.7±0.5	0.97	+68.1%
*E*_time_+PhysLoss	2.51±0.08	3.1	4.01±0.12	87.2±0.5	7.52±0.19	3.96±0.11	86.1±0.4	1.03	+59.8%
OpenGLA	2.34±0.06	2.8	3.61±0.11	89.4±0.5	6.42±0.18	3.61±0.10	88.9±0.4	1.09	+54.2%
**Rollout-only (matched)**	2.78±0.09	3.5	4.49±0.14	83.9±0.7	8.22±0.25	3.72±0.13	84.2±0.6	1.00	+61.5%
TypedGNN-OPF (Sup.-only)	–	–	3.08±0.10	93.2±0.4	–	–	–	0.88	–
DiffFlow (Sup.-only)	–	–	2.97±0.08	94.1±0.3	6.08±0.16	–	–	1.21	–
LUMINA (Sup.-only)	–	–	2.74±0.07	95.0±0.3	–	3.12±0.09	–	1.18	–
**PhysBridge**	1.73±0.04	**1.8**	2.21±0.05	96.3±0.2	4.21±0.11	1.84±0.05	91.6±0.3	1.24	**+27.7%**

**Transfer Gap Under Response Pretraining and Local Refinement:** The results in Table 5 show PhysBridge with the lowest OPF gap (2.21±0.05%) and highest feasibility (96.3±0.2%) in the transfer setting. The matched rollout-only comparison is the key controlled result: using the same backbone and source corpus, response-oriented pretraining plus local refinement reduces OPF gap by 50.8%, reduces voltage MAE by 48.8%, reduces LMP MAE by 50.5%, and raises fault macro F1 by 7.4 percentage points. A 24-hour moving-block bootstrap gives Holm-adjusted *p* < 0.01 for all five task metrics. The table also mixes multi-task pretrained models with supervised-only task specialists; gains over LUMINA and TypedGNN-OPF therefore remain task-relevant comparisons rather than architecture-only evidence.

**Label Efficiency Dynamics:** The label-budget results in Table 6 reveal a nonlinear relationship between label availability and the utility of solver-guided pretraining. At 1% labels, PhysBridge remains ahead of Scratch, OpenGLA, and the matched rollout-only model; as the label ratio approaches 100%, the gap narrows. This pattern suggests that response-oriented pretraining acts primarily as a data-efficiency multiplier, especially when paired with local refinement around the operating point.

**Table 6:** Label-efficiency results on IEEE 300-bus (mean ± standard deviation). Each entry reports OPF cost gap (%) / contingency/fault event macro F1 (%).

Method	1% labels	5% labels	10% labels	100% labels
Scratch	8.91±0.30 / 71.3±1.0	6.44±0.22 / 78.9±0.7	5.36±0.18 / 82.8±0.6	3.11±0.12 / 91.7±0.3
OpenGLA	6.24±0.21 / 78.4±0.8	4.55±0.14 / 84.9±0.5	3.61±0.11 / 88.9±0.4	2.34±0.06 / 94.0±0.2
Rollout-only (matched)	7.42±0.25 / 74.0±0.9	5.38±0.18 / 80.1±0.7	4.49±0.14 / 84.2±0.6	2.78±0.09 / 92.2±0.3
**PhysBridge**	3.62±0.11 / 84.9±0.7	2.68±0.07 / 89.2±0.4	2.21±0.05 / 91.6±0.3	1.73±0.04 / 95.2±0.2

**Per-Class Event Identification Audit:** To make the contingency/fault task interpretable, the revised analysis reports class-level precision, recall, F1, and false-alarm rate in Table 7. Table 8 provides the corresponding confusion matrix for an independently trained seed whose macro F1 is 91.53%, within the three-seed result reported in the main comparison.

**Table 7:** Per-class contingency/fault event identification results on the IEEE 300-bus stressed suite for the seed reported in Table 8. False-alarm rate is computed one-vs-rest as *FP*/(*FP* + *TN*).

Class	Share (%)	Precision	Recall	F1	False-alarm rate
No event	70.0	0.9662	0.9700	0.9681	0.0792
Line outage/switching	10.0	0.9240	0.9180	0.9210	0.0084
Generator trip	6.0	0.9070	0.8990	0.9030	0.0059
Bus load/renewable disturbance	8.0	0.9190	0.9110	0.9150	0.0070
Combined *N*-2 event	6.0	0.8760	0.8630	0.8695	0.0078

**Table 8:** Confusion matrix for contingency/fault event identification on the 420,000-window IEEE 300-bus stressed test suite. Rows denote true classes and columns denote predicted classes. This independently trained seed obtains a macro F1 of 91.53%; its class-level metrics are reported in Table 7.

True \ Pred.	No event	Line/switch	Gen. trip	Load/RES	Combined *N*-2	Total
No event	285,180	2,500	2,100	2,000	2,220	294,000
Line outage/switching	2,297	38,556	33	400	714	42,000
Generator trip	1,945	400	22,655	200	0	25,200
Bus load/renewable disturbance	2,562	226	57	30,610	145	33,600
Combined *N*-2 event	3,176	45	133	98	21,748	25,200
**Total**	**295,160**	**41,727**	**24,978**	**33,308**	**24,827**	**420,000**

### 5.2 Robustness and generalization boundaries under distributional shifts

To address practical deployment concerns, we depart from idealized clean benchmarks and subject the models to a unified stress test encompassing both *measurement corruptions* (noise, delays) and *domain shifts* (topology scaling, generation mix changes). The results are aggregated in Table 9.

**Table 9:** Performance under measurement corruptions and structural shifts on IEEE 300-bus (mean ± standard deviation). Values denote OPF cost gap (%) / fault macro F1 (%); “–” indicates that the method does not expose a compatible fault-classification output.

Shift Category	Severity	OpenGLA	DiffFlow	PhysBridge
*Measurement Corruptions*	Bad Data (8% sensors)	6.13±0.21 / 79.1±0.8	5.88±0.19 / –	3.81±0.12 / 87.2±0.5
	Heavy-tailed Noise (Student-*t*)	4.85±0.16 / 83.2±0.6	4.46±0.14 / –	3.02±0.09 / 89.1±0.4
	Telemetry Delay (2 steps)	4.37±0.14 / 84.7±0.5	4.11±0.13 / –	2.89±0.08 / 90.4±0.4
	Combined (Bad Data + *N*-2)	6.91±0.24 / 76.4±1.0	6.47±0.22 / –	4.62±0.16 / 84.1±0.7
*Structural Domain Shifts*	Bus-ratio gap 3×–10×	2.87±0.08 / 90.9±0.4	–	1.57±0.04 / 94.1±0.2
	Bus-ratio gap >10×	3.61±0.11 / 88.9±0.4	–	2.21±0.05 / 91.6±0.3
	Renewable Mismatch (+30 pp)	5.28±0.17 / 82.1±0.7	–	3.74±0.11 / 86.4±0.6

**Resilience via Local Refinement:** PhysBridge consistently outlasts baselines under heavy-tailed noise and bad data injection. From a physical perspective, generic graph models like OpenGLA tend to overfit to localized noisy node features, causing ripple effects in predicted power flows. In contrast, the interior-point unrolling within PhysBridge actively penalizes predictions that violate global nodal power balance. This refinement-driven correction helps filter out non-physical anomalies (e.g., physically impossible voltage spikes caused by sensor errors) before they corrupt the final task heads.

**Explicit Generalization Boundaries:** Despite its robustness, Table 9 deliberately maps out the model’s limitations to prevent overclaiming. We observe a monotonic degradation as the topological distance and generation mix diverge from the pretraining distribution. Specifically, when the bus-ratio gap exceeds 10× and renewable penetration shifts by + 30 percentage points, the OPF Cost climbs to 3.74% and event F1 drops to 86.4%. Similarly, the “Combined Contingency” (*N*-2 failure coupled with bad data) inflicts severe damage across all models, proving that PhysBridge is not a panacea. The graph transformer’s positional embeddings still struggle with massive zero-shot structural extrapolation, defining a clear boundary: PhysBridge facilitates transfer to *moderately* larger, related grids, but cannot unconditionally generalize to entirely disparate power networks without further architectural scaling.

### 5.3 Mechanistic Insights: Ablations, Stability, and Overheads

We isolate the contributions of specific algorithmic designs and quantify the unavoidable computational tax of physics-informed AI.

**Ablation of Learning Objectives:** Table 10 separates access to the source corpus, response-oriented supervision, local refinement, and token composition. The matched rollout-only model receives the same 21.04 million source-topology pairs but removes both response targets and bridge refinement. A second “No PDR-Bridge” row retains response supervision while bypassing local refinement. Their difference shows how response targets remain useful without the solver layer, whereas their common gap to the full model quantifies the additional benefit of local physics-guided refinement. Removing ${ℒ}_{resp}$ increases OPF gap from 2.21% to 3.32% and LMP MAE from 1.84 to 2.68 $/MWh. Removing projection-dual or sensitivity tokens causes smaller but consistent degradation. Following the reviewers’ request, Table 11 adds the complementary rollout-only plus PDR-Bridge and compute-matched rollout-only baselines needed to isolate bridge-only, response-only, and compute-budget effects.

**Table 10:** Ablation study on IEEE 300-bus with 10% labels (mean ± standard deviation). All rows except Scratch use the same source-topology corpus and downstream adaptation protocol.

Variant	OPF Gap (%)	Feas. (%)	SE V-MAE (10^–3^ p.u.)	LMP MAE	Event F1 (%)
Rollout-only, no bridge	4.49±0.14	83.9±0.7	8.22±0.25	3.72±0.13	84.2±0.6
Response targets, no PDR-Bridge	4.14±0.12	84.4±0.6	8.79±0.27	3.19±0.10	84.9±0.5
Projection-dual tokens removed	2.97±0.08	92.5±0.4	5.94±0.17	2.36±0.07	89.0±0.4
Sensitivity tokens removed	2.80±0.07	93.5±0.3	5.58±0.16	2.26±0.06	89.7±0.4
Binding-set loss removed	2.69±0.07	92.1±0.4	4.98±0.14	2.11±0.06	90.2±0.3
${ℒ}_{resp}$ removed	3.32±0.09	90.7±0.5	5.26±0.15	2.68±0.08	87.1±0.5
${ℒ}_{roll}$ removed	2.56±0.06	94.7±0.3	5.01±0.13	2.07±0.06	90.5±0.3
**PhysBridge (full)**	2.21±0.05	96.3±0.2	4.21±0.11	1.84±0.05	91.6±0.3

**Table 11:** Factorial ablation results for the revised analysis. All variants use the same source-data exposure unless explicitly compute-matched.

Variant	Rollout loss	Response targets	PDR-Bridge	Pretrain updates	A100-hours	OPF/Event metrics
Rollout-only, no bridge	Yes	No	No	100K	33.2±1.1	4.49±0.14 / 84.2±0.6
Rollout-only + PDR-Bridge	Yes	No	Yes	100K	35.9±1.2	3.86±0.11 / 85.4±0.6
Response targets, no PDR-Bridge	Yes	Yes	No	100K	34.7±1.0	4.14±0.12 / 84.9±0.5
Compute-matched rollout-only	Yes	No	No	126K	41.8±1.6	4.31±0.13 / 84.8±0.6
**PhysBridge**	Yes	Yes	Yes	100K	41.8±1.6	2.21±0.05 / 91.6±0.3

The factorial comparison shows that access to the large corpus alone is insufficient: response supervision without the bridge improves LMP prediction relative to rollout-only, while the bridge-only and compute-matched controls distinguish the two mechanisms.

**OPF Cost Accounting and Certified Recovery:** Because the bridge corrects states around a fixed predicted dispatch, aggregate cost gaps can be misleading when infeasible predictions are included. Table 12 therefore adds a cost-accounting audit that separates feasible-only cost, penalized cost, constraint violations, and recovery by a certified MATPOWER AC-OPF solve initialized from the learned output.

**Table 12:** OPF cost-accounting and certified AC-OPF recovery audit on IEEE 300-bus. For the maximum power-flow residual and maximum bound-violation columns, the maximum is taken over test samples within each seed and the table reports the mean ± standard deviation across the three seeds.

Method	Feasible-only cost gap (%)	Penalized cost gap (%)	Max PF resid.	Max bound viol.	AC-OPF recovery (%)	Recovery iters
Scratch	5.02±0.17	8.37±0.28	$1.92\pm 0.15\times 10^{-3}$	$4.18\pm 0.31\times 10^{-2}$	89.8±0.8	26.4±1.3
Rollout-only, no bridge	4.17±0.13	6.71±0.21	$1.43\pm 0.12\times 10^{-3}$	$3.32\pm 0.25\times 10^{-2}$	93.1±0.6	22.8±1.1
Rollout-only + PDR-Bridge	3.57±0.10	5.12±0.17	$0.96\pm 0.09\times 10^{-3}$	$2.41\pm 0.18\times 10^{-2}$	96.7±0.4	18.6±0.9
Response targets, no PDR-Bridge	3.86±0.11	5.89±0.19	$1.18\pm 0.10\times 10^{-3}$	$2.87\pm 0.20\times 10^{-2}$	95.0±0.5	20.4±1.0
**PhysBridge**	2.05±0.05	2.31±0.06	$0.42\pm 0.04\times 10^{-3}$	$0.84\pm 0.07\times 10^{-2}$	99.6±0.1	13.7±0.6

**Projection Duals versus OPF LMPs:** Table 13 empirically illustrates the distinction derived in Section 3. Directly interpreting the projection equality multiplier $\lambda^{P}$ as an LMP gives poor price accuracy. Training an LMP head without projection-dual features is substantially better, while the full head that consumes projection-dual and sensitivity tokens gives the lowest MAE and highest rank correlation. This pattern is consistent with treating projection duals as informative local-stress features rather than market prices.

**Table 13:** Validation of the relationship between projection-dual tokens and OPF LMP targets on IEEE 300-bus (mean ± standard deviation).

Price estimator	LMP MAE ($/MWh)	Spearman ρ
Direct projection dual $\lambda^{P}$	5.72±0.17	0.61±0.02
LMP head without projection-dual tokens	2.37±0.07	0.84±0.01
**Full LMP head**	1.84±0.05	0.91±0.01

**Latency, Failure Modes, and Compute Limits:** The primary critique against embedding differentiable solvers is numerical instability and latency. As detailed in Table 14, executing *K* = 3 interior-point steps on a single NVIDIA A100 80GB GPU requires 12.4 ms on the 300-bus system, approximately 24% more than the normalized Scratch runtime in Table 5. The full pretraining budget is 41.8±1.6 A100-hours, compared with 33.2±1.1 A100-hours for the matched rollout-only model. Under stressed conditions, 4.6% of test windows trigger fallback because the residual remains above tolerance or the KKT Jacobian becomes ill-conditioned. These windows are not removed during training: they contribute through the explicit fallback path, health bit $m_{t}$, and bounded stability penalty.

**Table 14:** Forward/backward runtime profile on a single NVIDIA A100 80GB GPU.

Component	Fwd (ms)	Bwd (ms)	Mem (GB)	% of Fwd
*E*_time_ (Mamba)	2.1	3.4	1.2	16.9%
*E*_graph_ (Transformer)	3.2	5.7	2.8	25.8%
PDR-Bridge	4.8	8.3	3.1	38.7%
Task head	0.4	0.6	0.3	3.2%
Other	1.9	1.8	0.6	15.3%
Total	12.4	19.8	8.0	100%

The PDR-Bridge is the most expensive module, accounting for roughly two-fifths of forward cost because of interior-point unrolling and Jacobian-related linear solves. Even so, total latency remains below the 1–5 s operational cycle commonly assumed for dispatch and monitoring applications.

To quantify the downstream impact of unsuccessful local refinement, Table 15 separates stressed windows by bridge health.

**Table 15:** Disaggregation by bridge health on the 420,000-window IEEE 300-bus stressed suite (mean ± standard deviation). Fallback = True corresponds to $m_{t}=0$.

Subset	Windows	Share	OPF Gap (%)	Feas. (%)	V-MAE (10^–3^ p.u.)	LMP MAE	Event F1 (%)
Fallback = False ($m_{t}=1$)	400,680	95.4%	2.12±0.05	98.9±0.1	4.05±0.10	1.76±0.04	92.0±0.3
Fallback = True ($m_{t}=0$)	19,320	4.6%	4.08±0.16	42.4±1.3	7.53±0.28	3.50±0.14	83.3±0.8
**All stressed windows**	420,000	100%	2.21±0.05	96.3±0.2	4.21±0.11	1.84±0.05	91.6±0.3

The disaggregated results show that fallback windows are materially harder across every task, with the largest deterioration in feasibility. In the failure audit, combined bad-data and *N*-2 events account for 52% of fallback activations, isolated bad-data bursts for 24%, topology mismatch for 15%, and other heavily loaded cases for 9%. The explicit health bit lets downstream heads condition on this reliability state, but it does not remove the need for operator-side validation or a certified solver. Hyperparameter sweeps place *K* = 3 near the knee of the trade-off curve; increasing to *K* = 5 changes OPF gap from 2.21% to 2.14% while increasing latency and memory pressure.

**Hyperparameter Sensitivity:** We also retain the main hyperparameter sweeps for completeness. The results in Table 16 show that PhysBridge is reasonably stable across a broad range of settings. In particular, the selected defaults $\left(\beta =0.5,K=3,W=288,d_{b}=16\right)$ lie on or near the best trade-off frontier. Increasing *K* beyond 3 yields only marginal gains in OPF gap, consistent with the runtime discussion above.

**Table 16:** Sensitivity to key hyperparameters on IEEE 300-bus with 10% labels. Values report OPF cost gap (%, mean ± standard deviation).

Hyperparameter	Values	Results
β	0.1, 0.25, 0.5, 1.0, 2.0	2.37±.07, 2.28±.06, 2.21±.05, 2.26±.06, 2.41±.07
*K* (IP steps)	1, 2, 3, 4, 5	2.63±.08, 2.34±.06, 2.21±.05, 2.17±.05, 2.14±.05
*W* (window)	72, 144, 288, 576, 1152	2.49±.08, 2.31±.06, 2.21±.05, 2.18±.05, 2.17±.05
$d_{b}$ (perturb.)	4, 8, 16, 32, 64	2.43±.07, 2.29±.06, 2.21±.05, 2.20±.05, 2.23±.06

## 6 Discussion

The matched rollout-only comparison supports a narrower attribution than the unmatched specialist comparison. Corpus size alone does not reproduce the full pattern: response targets improve task-relevant representation learning, while local refinement and bridge tokens supply additional gains in feasibility, state estimation, and LMP prediction. The label-efficiency table further suggests that the benefit is largest when target labels are scarce. By contrast, the supervised-only OPF references remain useful task-level anchors but do not identify architecture-only effects because they do not receive the same pretraining data.

The numerical behavior of the bridge is equally important. Since local AC refinement is nonconvex, stability must be treated as an empirical property rather than a theorem. The fallback split shows that the aggregate result can conceal a substantially weaker tail: the 4.6% fallback subset has roughly twice the OPF gap and far lower feasibility than successful-refinement windows. Projection-dual variables also should not be interpreted as market prices. Their direct LMP accuracy is poor, but they improve a supervised LMP head when combined with primal and sensitivity features. PhysBridge is therefore best understood as a learning-augmented representation and warm-start layer, not as a replacement for certified optimization or operator validation.

Several limitations remain. First, the benchmark is built from IEEE standard test cases and solver-generated operating points rather than utility field records, and it omits protection actions, three-phase imbalance, detailed inverter controls, and cyber-physical attack logic. Second, the task-specialist comparison remains partially unmatched because these methods do not receive the same pretraining corpus. Third, three seeds provide an initial estimate of training variability, but additional seeds and per-window hierarchical analysis would further strengthen the statistical evidence. Finally, validation on utility data or a high-fidelity digital twin remains necessary before deployment.

## 7 Conclusions

We presented PhysBridge, a response-oriented pretraining framework with physics-guided local refinement for cross-topology grid operations. The PDR-Bridge uses safeguarded differentiable refinement, residual screening, fallback recovery, and primal, projection-dual, sensitivity, and health tokens. Its local problem refines state feasibility with the predicted control fixed; it is not a globally convergent OPF solver, and its projection multipliers are not direct LMP estimates.

The evaluation includes a matched rollout-only baseline, uncertainty reporting, label-efficiency analysis, projection-dual validation, runtime accounting, and fallback-disaggregated metrics. Across three runs on IEEE 300-bus with 10% labels, PhysBridge reports a 2.21±0.05% OPF gap, 96.3±0.2% feasibility, 1.84±0.05 $/MWh LMP MAE, and 91.6±0.3% fault macro F1. The matched comparison separates the effect of response targets and local refinement from access to a large source corpus.

These results support PhysBridge as a learning-augmented representation and warm-start framework, but they do not establish deployment readiness. Field validation and stronger guarantees for solver reliability remain necessary.
